# Hypergastrinemia

**DOI:** 10.1093/gastro/gov004

**Published:** 2015-02-18

**Authors:** Sunil Dacha, Mohammed Razvi, Julia Massaad, Qiang Cai, Mohammad Wehbi

**Affiliations:** Internal Medicine, Emory University, Atlanta, GA, USA

**Keywords:** hypergastrinemia, carcinoid, *helicobacter pylori*

## Abstract

Gastrin is an important hormone of the digestive system, which assists gastric acid secretion. It may be pathologically elevated in conditions such as Zollinger-Ellison syndrome, or due to common medications such as proton pump inhibitors. In this review we provide an overview of the pathophysiology and medical causes of hypergastrinemia, diagnostic testing and clinical consequences of chronic hypergastrinemia.

## Introduction

Gastrin is one of the most important and clinically relevant hormones of the digestive system and has been studied extensively for the past decade. It is released by the G cells of the antrum of the stomach. Besides assisting in the stimulation of gastric acid secretion, it also facilitates proliferation of the gastric epithelial cells, tissue remodeling, and angiogenesis [1]. Abnormal gastrin production occurs in some clinical and diseased states, a condition known as hypergastrinemia and defined by a Gastrin level greater than 100–150 pg/ml [2].

It is necessary to monitor gastrin levels in a few conditions, including (i) refractory or recurrent peptic ulcer disease (PUD) in the absence of non-steroidal anti-inflammatory drugs or *helicobacter pylori* (*H. pylori*) infection, (ii) PUD in unusual locations (e.g. beyond the duodenal bulb), (iii) PUD with concurrent endocrinopathies, (iv) gastroesophageal reflux disease (GERD) refractory to proton pump inhibitors (PPIs) and/or with distal esophageal strictures, (v) presence of prominent rugal folds seen on upper endoscopy, (vi) chronic secretory diarrhea and (vii) gastric carcinoids. In these clinical conditions with abnormal gastrin production, it is therefore important to check for abnormal gastrin levels and to look for the source, if elevated. It is equally important for physicians and other practitioners to be aware of the clinical conditions in which gastrin monitoring is required and the implications of the results for the individual patient.

There has recently been immense interest in the pathophysiology of gastrin, due to extensive use of proton pump inhibitors (PPIs) and the resulting hypergastrinemia. PPI'S are available over the counter and are used indiscriminately for treating dyspepsia, acid reflux, gastritis and peptic ulcers without appropriate indication. *H. pylori* infection can, in general, also raise gastrin levels and it has become one of the most common reasons for hypergastrinemia. Some studies have raised concerns about the associated progression of colorectal cancer and occurrence of neoplasms of the stomach. The validity of these concerns is still under close scrutiny and is being studied extensively. Enterochromaffin-like (ECL) cell hyperplasia, and increased *H. pylori*-induced gastric atrophy has been noted in some recent studies, but their link to more severe diseases is yet to be determined. We present a review of the pathophysiology of gastrin secretion, as well as some known causes and implications of hypergastrinemia.

## Pathophysiology

Stomach acid is produced by parietal cells that line the stomach wall. These cells have proton pumps which move hydrogen ions from the inside the parietal cell into the stomach lumen against a concentration gradient. These pumps secrete acid in response to three neurohumoral signals: (i) acetylcholine, a neurotransmitter that is released by the vagal nerve endings, (ii) gastrin, a local hormone produced by G cells in the antrum, and (iii) histamine, a biologically active chemical produced by ECL-cells in the stomach wall.

**Figure 1.**
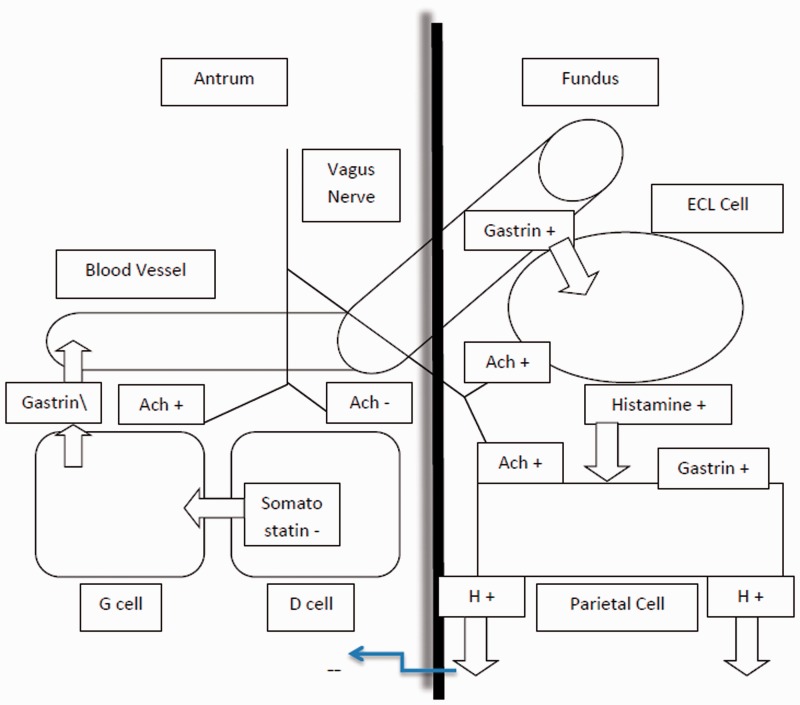
Physiology of gastric acid secretion. Histamine, acetylcholine and gastrin effect on gastric acid secretion is shown (Reference: Katzung BG, Masters SB, Trevor AJ. Basic & Clinical Pharmacology, 11^th^ edition. http://www.accessmedicine.com).

Gastrin stimulates the parietal and pepsin cells, increases gastric mucosal blood flow, and has a trophic effect on the gastric, duodenal and colonic mucosa [3]. Its main roles include food-stimulated gastric acid secretion and trophic effects on the ECL-cells [4, 5]. Figure 1 demonstrates the effect of gastrin on parietal cells and its role in acid secretion.

Gastrin is initially synthesized as pro-gastrin, a pro-hormone, which is cleaved into gastrin molecules of various lengths [6]. This is eventually secreted into the bloodstream with various degrees of affinity to gastrin receptors. There are two established types of gastrin receptors: cholecystokinin (CCK)-A and CCK-B. Circulating gastrin stimulates the CCK-B receptors on the basolateral membrane of the parietal cells, directly causing gastric acid secretion. Gastrin is released by the G cells in response to food intake. The proteins and amino acids contained in food are the primary triggers for gastrin secretion [7]. Gastrin release and formation by G cells is stimulated by gastrin-releasing peptide and inhibited by somatostatin secreted from D cells within the stomach lining.

The pH of the stomach also has a regulatory role in gastrin release, whereby fasting or increased acidity will inhibit gastrin, while decreased acidity in the stomach will stimulate gastrin [7]. Gastric acid can also be released indirectly when histamine, released from the ECL-cells, binds to the H_2_ receptors on the parietal cells [4, 6].

## Causes

There are two major categories of hypergastrinemia: those that are associated with acid hypersecretion and those that are not. Appropriate gastrin secretion occurs with neutral pH, while unopposed gastrin secretion in the presence of an already acidic pH is an inappropriate response, as in gastrin-producing tumors or gastrinomas [6]. In patients with gastrinomas, high serum gastrin concentrations are responsible for acid hypersecretion, which induces ulcers in the stomach. In patients with duodenal ulcers, gastrin tends to be elevated in the bloodstream and there is an increased gastrin response to feeding, and an impairment of the ability of low pH to inhibit gastrin [8]. Table 1 summarizes the various causes of hypergastrinemia in relation to acid production.

**Table 1.** Causes of hypergastrinemia in relation to acid production

Decreased acid production	H2 blocker or PPI therapy
	*H. pylori* infection
	Atrophic corporal gastritis
	Renal failure
	Vagotomy
Normal or increased acid production	Zollinger-Ellison syndrome
	Retained antrum syndrome
	Antral G cell hyperplasia
	Gastric outlet obstruction

The most important step in the evaluation of hypergastrinemia is the determination of gastric acid production [9]. The clinical indications for gastrin secretion analysis are relatively limited and most hospitals no longer possess the resources to perform this test. In hypergastrinemic conditions, however, quantification of gastric acid secretion can determine whether or not the elevated gastrin is an appropriate response, and will therefore influence further test and therapy.

The basal acid output (BAO) is the sum of four 15-minute samples of acid secretion expressed as milli-equivalents per hour. The upper limits of normal BAO for men and women are 10 and 6 mEq/hr, respectively. Nowadays BAO is rarely measured and a rough estimate of gastric acidity is made by a gastric aspirate during endoscopy; a pH of <4 will exclude the possibility of anacidity [9, 10].

### Appropriate hypergastrinemia: decreased acid production

#### H2 blockers and proton pump inhibitors

Since the development of PPIs in the late 1980s, the significance of hypergastrinemia has become a worldwide topic of research and clinical concern [5, 6]. Since gastrin secretion is inhibited by gastric acidity, medications like H2 blockers and PPIs tend to cause hypergastrinemia. PPIs directly inhibit hydrogen ion exchange and inhibit secretion in response to all stimulatory agents, by irreversibly blocking the proton pump [11]. H2 receptor blockers only block histamine, leaving gastrin and acetylcholine as potential stimuli. PPIs are better inhibitors of gastric acid secretion than H2 receptor blockers and are therefore associated with higher gastrin levels. Gastrin elevation in these conditions is usually modest, ranging from 200–400 pg/ml [10], with even more severe elevations reported in the literature [12–15]. Studies lasting up to 5 years have shown that plasma gastrin levels generally peak (10–20% above baseline) in the first four months of treatment with PPIs, and stabilize without further increase thereafter [16, 17]. The concern over this degree of hypergastrinemia relates largely to the trophic effect of gastrin on the oxyntic mucosa of the stomach and the subsequent development of gastric carcinoids in rat models [5]. It has been suggested that gastric carcinoids develop following progressive changes caused by gastrin stimulation, such as increasing hyperplasia of ECL-cells to dysplasia and carcinoid formation [18]. Multiple studies have shown the hyperplastic effects of gastrin on the gastric mucosa in the setting of antacid medication in rats, but the evidence for dysplasia or carcinoid development in humans is yet to be ascertained [19, 20]. In contrast to humans, rats have a higher density of gastrin ECL-cells and a greater gastric response to hypochlorhydria. Studies on other animals—such as mice, dogs and hamsters—have not produced the same results as in rats [21].

Cessation of the offending drugs will usually reverse the hypergastrinemia within 5–7 days. Monitoring gastrin levels in patients on long-term anti-secretory medications is not currently recommended, as it is costly and offers little clinical benefit [22]. There is significant individual variation in gastrin levels among patients on PPI therapy, and clinically significant gastrin elevations are not seen in all these [22]. In addition, there is no clear evidence of carcinoids in patients treated with PPIs for 5–7 years, suggesting that PPI treatment has a low probability of causing carcinoids in humans [23].

#### Atrophic corporal gastritis with and without pernicious anemia

Chronic atrophic corporal gastritis type A (ACG-A) is an autoimmune inflammatory disease characterized by the destruction of the gastric glands and parietal cell mass. This causes a block in the negative feedback mechanism of somatostatin and results in hypergastrinemia. ACG-A is associated with the highest frequency of ECL-cell carcinoids in humans [24]. In severe cases, such as in pernicious anemia, complete destruction of the parietal cell mass leads to achlorhydria, increased antral pH and hypergastrinemia (which can be very severe—greater than 1000 pg/ml) [25]. In response to chronically elevated gastrin levels, ECL cell hyperplasia is induced and carcinoids may develop through the hyperplasia–dysplasia–neoplasia sequence. The development of a neoplastic phenotype is probably related to ECL proliferation in this physiological setting [26, 27].

#### Heliobacter pylori infection

*H. pylori* infection is one of the most common etiologies of hypergastrinemia. This gram-negative organism can damage the gastric glands and parietal cells via infiltration, which leads to decreased acid production and secondary hypergastrinemia; however, gastrin levels are usually modest in this form of gastritis, and carcinoids are uncommon in this setting [28]. Asymptomatic patients with *H. pylori* infections have elevated serum gastrin concentrations relative to controls, despite having the same gastric acid output [29]. It has been suggested that the elevation of antral surface pH is caused by increased production of gastric ammonia, decreased somatostatin content in D cells, and mucosal cell injury [30]. The site of colonization by this bacterium is important, with antral infections causing peptic ulcer disease and gastric corpus infections more likely to cause gastric atrophy and hypochlorhydria [1]. Chronic ACG type B is a chronic inflammatory disease of the stomach due to infection with *H. pylori*. It is important to note, however, that not all *H. pylori* infections will result in atrophic changes and there have been reports of hypergastrinemia and hyperchlorhydria secondary to *H. pylori* infection that is independent of atrophic gastritis [31]. Kuipers *et al.* reported that atrophic gastritis developed more frequently with chronic PPI treatment in patients who are *H. pylori-*positive than in patients treated with fundoplication [32]. In *H. pylori-*negative patients this development was not seen. This may imply that the development of atrophic gastritis is a major prognostic factor for increasing the risk of *H. pylori* infection’s association with gastric cancer [33]. Hypergastrinemia in the setting of chronic *H. pylori* infection is most strongly associated with the development of gastric adenocarcinoma [1]. On the other hand, hypergastrinemia in the absence of *H. pylori* is mostly associated with gastric neuroendocrine tumors [34].

#### Renal failure

End-stage renal disease (ESRD) patients have higher than normal circulating levels of gastrin, probably due to decreased renal clearance of gastrin, increased gastric G cell density, and decreased inhibition secondary to diminished somatostatin levels [35]. With the increasing concern over the trophic effects of gastrin on the gastric mucosa, the hypergastrinemia in this subgroup of patients could be contributing to the hypertrophy of the gastro-intestinal (GI) tract and occurrence of gastritis frequently noted in ESRD patients [36, 37]. *H. pylori* colonization/infections are very common in ESRD. Patients with ESRD tend to have an even more potentiated hypergastrinemia than those without. The majority of these patients will have concurrent hypochlorhydria along with hypergastrinemia, although a small subset of patients may also have normal acid secretion or even hyperchlorhydria.

#### Vagotomy

In the past, surgical therapy for peptic ulcer disease included truncal, selective, or superselective vagotomy without antrectomy [38]. This approach decreased the muscarinic stimulation of parietal cells by acetylcholine and hence decreased acid secretion, which results in higher antral pH and triggers gastrin secretion. Gastrin elevation in this condition is usually mild (200 pg/ml). The clinical significance of hypergastrinemia in this setting is negligible [39].

### Inappropriate hypergastrinemia: normal or increased acid production

#### Zollinger-Ellison syndrome

Zollinger-Ellison syndrome (ZES) is a syndrome characterized by hypersecretion of gastrin from gastrinomas—a type of neuroendocrine tumor—which can lead to refractory peptic ulcers in the upper gastro-intestinal tract. ZES is sporadic in the majority of cases, but it is also the most common functional pancreatic endocrine tumor syndrome in patients with multiple endocrine neoplasia type-1 syndrome (MEN-1) [40]. MEN-1 is found in 20–38% of all patients with gastrinomas. Gastrinomas causes severe, unopposed gastrin elevations (>2000 pg/ml, although it can have a wide range of 300–2000 pg/ml) [2]. These tumors are typically multiple and localized to the pancreas (80%) or the duodenal wall (20–30%) [41]. They may be malignant (18–60%) and lead to multiple ulcerations throughout the GI tract [41].

ZES can present as a constellation of symptoms that reflect the hypergastrinemia associated with this disease. Common features of ZES include diarrhea, multiple relapsing ulcers in atypical locations throughout the GI tract, and non-beta cell pancreatic tumors [42]. The association between hypergastrinemia and gastric carcinoids is well documented in the literature, with type II carcinoids being a direct consequence of MEN-1. The diagnosis of gastrinoma can be confirmed with a secretin stimulation test, with an increase in circulating gastrin levels of >200 pg/ml above baseline after intravenous administration of 1–2 µg/kg of body weight of secretin [43, 44]. Studies on patients with sporadic ZES not associated with MEN-1, demonstrate that gastric carcinoids occur rarely in these patients (with incidence <1%) whereas, in ZES- associated with MEN-1, gastric carcinoids can occur in as many as 13–43% [45]. This difference in the occurrence of carcinoids is observed despite the chronic hypergastrinemia present in both types of ZES.

These results, along with other studies, have led us to believe that hypergastrinemia alone is only ECL-growth-promoting and not sufficient for carcinoid development without other necessary factors [46].

#### Retained antrum syndrome

Recurrence of peptic ulceration is uncommon after surgical treatment unless there are identifiable causes, including gastrinoma, NSAID intake or an incomplete vagotomy. Recurrence may also result from incomplete excision of the gastric antrum from the duodenum (retained antrum) during surgery. Gastrin elevation in this entity is only modest, and hypergastrinemia is reversible with excision of the retained antral remnant [47]. With the advancement of surgical techniques in the past few years, retained gastric antrum syndrome has been viewed as a rare entity due to careful excision of the antrum [48].

#### Antral G-cell hyperplasia

Pseudo-Zollinger Ellison Syndrome (Ps-ZES), or antral G-cell hyperplasia, is a rare entity characterized by a marked hypergastrinemia. It is associated with increase in the number of G cells, poor response to secretin stimulation test, and absence of gastrinoma in the pancreas or duodenum. This does not fit the classic triad that defines ZES. A few case reports of peptic ulcer disease secondary to antral G-cell hyperplasia have been reported in the literature [49, 50]. Even though this is a rare disease, it should be considered in patients with Zollinger-Ellison syndrome without evidence of a gastrin producing tumor. Differentiation between the two may be difficult, but Ps-ZES tends to have lower gastrin levels than ZES [51]. A gastrin stimulation test using a Standard Test Meal will produce a three-fold rise in antral gastrin in Ps-ZES compared to only 40% increase in ZES [52]. Differentiating these two diagnoses is clinically important to avoid subjecting the Ps-ZES to unnecessary surgical procedures.

#### Gastric outlet obstruction

Chronic gastric outlet obstruction results in antral distension, which initiates local and central cholinergic reflexes, causing release of acetylcholine. Acetylcholine then stimulates parietal cells to produce hydrochloric acid and interacts with G-cells to enhance gastrin secretion [53].

## Diagnostic testing

Measuring serum gastrin level is a non-specific measure of hypergastrinemia, and one of the first steps in evaluating patients presenting with symptoms consistent with that disorder. Results may range from mild- to severely elevated gastrin levels based on etiology. Gastrin levels should be obtained while the patient is fasting and PPI therapy should, ideally, be terminated at least one week prior to testing to ensure accurate results [2]. There used to be concerns over the validity of measuring fasting serum gastrin levels due to poor antibody characterization of gastrin immunoassays of gastrin concentrations. Modern gastrin immunoassays now use antibodies that only react against active gastrin molecules, and proper sample dilutions are carried out in order to accurately place high gastrin concentrations on the gastrin-inhibition curves for sample analysis [2]. It may also be important to repeat fasting serum gastrin levels several times on different days, as levels can fluctuate even within the same patient, depending on the underlying etiology [2]. The majority of studies suggest fasting serum gastrin levels in hypergastrinemia occur at >100–150 pg/ml [2].

When the diagnosis of hypergastrinemia is suspected, confirmatory tests need to be performed to rule out various clinical entities. As mentioned earlier, elevated gastrin level can be appropriate with increased gastric luminal pH. These cases tend to be patients receiving anti-secretory medications or with *H. pylori* gastritis. It can also be elevated in more severe diseases, such as in retained antrum. The secretin-provocative test is most useful in patients with recurrent ulcer disease, severe reflux esophagitis, and chronic diarrhea associated with modest elevations of serum gastrin levels. A secretin infusion at 1–2 µg/kg will usually produce a marked increase in serum gastrin levels (>200 pg/ml) in patients with Zollinger-Ellison syndrome and is accepted as a confirmatory test only (sensitivity and specificity >90%) [54]. In addition, stomach pH testing can also aid in diagnosis; ZES patients have been shown to have pH of less than 2 [55].

In some cases, it may be detrimental to suspend PPI therapy for diagnostic testing of hypergastrinemia. It has been observed that ZES patients can have dangerous complications as a result of interrupting PPI therapy for the sake of diagnosis [56]. Complications can occur as early as 48 hours after suspension of therapy and this may be explained by diminished protective mechanisms against increased acid secretion while on PPI therapy, such as pancreatic bicarbonate secretion, coupled with the baseline increase in gastrin secretion in these patients [56]. In order to avoid sudden complications, it is necessary to slowly wean down the PPI therapy over an extended period of time prior to testing, so that the body’s natural protective mechanisms can properly counteract the increased acid secretion, and alternate antacid medication must be provided during the weaning process [57]. The benefits of establishing a formal diagnosis need to be balanced with the risks of temporarily discontinuing anti-secretory therapy.

## Consequences

It has been previously proposed that gastrin acts as a co-factor during gastric carcinogenesis in hypergastrinemic patients, particularly in the setting of *H. pylori* [58]. Gastrin’s potent trophic action on the ECL-cell has been well established. Histopathologically, gastrin is thought to cause carcinoids through a sequence of hyperplasia- dysplasia- neoplasia of the ECL cell [59]. In the three main human hypergastrinemic conditions, ACG, MEN-1 + ZES, and ZES (alone), ECL cell hyperplasia develops only in the first two cases [60]. This implies that hypergastrinemia is necessary, but not sufficient alone, in instigating carcinoids. Given the large number of patients on antacid medications and the long duration of therapy, hypergastrinemia’s neoplastic potential has been of great interest in recent years. In patients treated with long-term acid suppression, the transformation of ECL cells to neoplastic lesions—namely carcinoids—has been observed in patients with atrophic gastritis and ZES in rats but not in humans [61, 62].

### Carcinoids

A direct causal relationship between acid-suppressive medications and carcinoid development in humans has not been established. Primary gastric carcinoids make up less than 1% of all gastric neoplasms [63]. The most frequent sites for carcinoids are in the GI tract (73.7%) and the broncho-pulmonary system (25.1%). Within the GI tract, most occur in the small bowel (28.7%), appendix (18.9%), and rectum (12.6%) [64]. Although rare, gastric carcinoids are known complications of prolonged severe hypergastrinemia [65] and ECL-cell hyperplasia is a precursor stage in the development of carcinoids.

There are three types of gastric carcinoids: types I and II that are associated with hypergastrinemia, and type III carcinoids, which are more sporadic lesions and are not a direct consequence of elevated gastrin levels [66].

1. The most common type of gastric carcinoids is type I (68–83%) [60], which is known to be associated with chronic atrophic gastritis type A. They are more common in women, tend to be multicentric, located in the fundus or body of the stomach, and have low metastatic potential [67].
2. Type II gastric carcinoids are associated with ZES and MEN-I and account for 5–10 % of all gastric carcinoids. They tend to be multiple, equal in males and females, and are more aggressive than type I, developing metastasis in 7–12% of cases [41]. Some of these lesions are gastrin-sensitive, and some surgeons suggest testing the tumor itself for gastrin sensitivity as a treatment option. One method of doing so is by biopsying the gastric *corpus mucosa* and neuroendocrine tumors before and after octreotide infusion, to see if there is a reduction in mRNA from secretory components of ECL cells. If gastrin sensitivity is positive, removing the gastrin source by antrectomy (in type I) or gastrinoma resection (in type II) should result in tumor regression [1]. On the other hand, more advanced tumors become large and mutated and no longer respond to gastrin, and may need a total gastrectomy.
3. Type III or sporadic carcinoids are the most aggressive of the three. They tend to be solitary lesions and are not associated with hypergastrinemia. They are more common in males and present with metastatic lesions more than 75% of the time [68]. Treatment of gastric carcinoids depends on the type of cancer and usually includes endoscopic or surgical resection. Somatostatin analogues are an effective and well tolerated therapy in minimizing the symptoms of the carcinoid syndrome. They decrease the symptoms of itching, diarrhea, bronchospasm, and cutaneous flushing, which are common problems in metastatic disease [69].

### Other Cancer Risks

Gastric adenocarcinoma has been associated with statistically significant elevation in serum gastrin levels compared with control patients for over 40 years [70], but the clinical significance of this link has yet to be determined [71, 72]. The association between hypergastrinemia and colorectal hyperplasia has been reported [73]. Hypergastrinemia might be trophic to colonic mucosa, but recent studies have shown that there is no link to colorectal cancer. Whether or not colorectal cancer cells possess gastrin receptors or play any role in tumor growth remains controversial. Epidemiological studies on patients with ZES and atrophic gastritis have shown no evidence of an increased incidence of colon cancer or polyps [74]. Two recent European case-control studies of a combined 10 000 patients with colorectal cancer, showed that PPI use of up to 7 years did not increase the risk for colorectal cancer [75, 76]. There are currently still no data to warrant surveillance for colorectal neoplasia in patients with hypergastrinemia.

Gastrin has been thought to stimulate the growth of other cancers. In epidemiological studies of ZES and atrophic gastritis patients, pancreatic, esophageal, and other hematologic cancers tend to be more prevalent [77–80]. Esophageal cancers have had a little more scrutiny after gastrin was found to increase COX-2 activation in Barrett’s esophagus. This activation of COX-2 was shown to inhibit apoptosis, stimulate cell proliferation, promote angiogenesis, and stimulate invasion by cancer cells. The esophagus also possesses CCK2 receptors, to which gastrin can bind, and promotes tumor growth [81]. Although not all studies agree on the role of gastrin in cancer development, the possible risk of cancer due to gastrin stimulation needs to be acknowledged.

## Conclusion

Hypergastrinemia is a common clinical entity that can be associated with hyper- or hypochlorhydria. Prolonged enterochromaffin cell exposure to gastrin can start a cascade of hyperplasia to neoplasia, with secondary tumors that have malignant potential. Clinicians should be aware of this disease and its long-term complications.

*Conflict of interest statement*: none declared.
